# A survey of energy drink consumption patterns among college students

**DOI:** 10.1186/1475-2891-6-35

**Published:** 2007-10-31

**Authors:** Brenda M Malinauskas, Victor G Aeby, Reginald F Overton, Tracy Carpenter-Aeby, Kimberly Barber-Heidal

**Affiliations:** 1 Department of Nutrition and Dietetics, East Carolina University, Greenville, North Carolina, USA; 2 Department of Health Education and Promotion, East Carolina University, Greenville, North Carolina, USA; 3 Department of Health, Physical Education, Recreation, and Dance, Virginia State University, Petersburg, Virginia, USA; 4 School of Social Work, East Carolina University, Greenville, North Carolina, USA

## Abstract

**Background:**

Energy drink consumption has continued to gain in popularity since the 1997 debut of Red Bull, the current leader in the energy drink market. Although energy drinks are targeted to young adult consumers, there has been little research regarding energy drink consumption patterns among college students in the United States. The purpose of this study was to determine energy drink consumption patterns among college students, prevalence and frequency of energy drink use for six situations, namely for insufficient sleep, to increase energy (in general), while studying, driving long periods of time, drinking with alcohol while partying, and to treat a hangover, and prevalence of adverse side effects and energy drink use dose effects among college energy drink users.

**Methods:**

Based on the responses from a 32 member college student focus group and a field test, a 19 item survey was used to assess energy drink consumption patterns of 496 randomly surveyed college students attending a state university in the Central Atlantic region of the United States.

**Results:**

Fifty one percent of participants (*n *= 253) reported consuming greater than one energy drink each month in an average month for the current semester (defined as energy drink user). The majority of users consumed energy drinks for insufficient sleep (67%), to increase energy (65%), and to drink with alcohol while partying (54%). The majority of users consumed one energy drink to treat most situations although using three or more was a common practice to drink with alcohol while partying (49%). Weekly jolt and crash episodes were experienced by 29% of users, 22% reported ever having headaches, and 19% heart palpitations from consuming energy drinks. There was a significant dose effect only for jolt and crash episodes.

**Conclusion:**

Using energy drinks is a popular practice among college students for a variety of situations. Although for the majority of situations assessed, users consumed one energy drink with a reported frequency of 1 – 4 days per month, many users consumed three or more when combining with alcohol while partying. Further, side effects from consuming energy drinks are fairly common, and a significant dose effect was found with jolt and crash episodes. Future research should identify if college students recognize the amounts of caffeine that are present in the wide variety of caffeine-containing products that they are consuming, the amounts of caffeine that they are consuming in various situations, and the physical side effects associated with caffeine consumption.

## Background

Energy drink consumption has continued to gain popularity since the 1997 debut of Red Bull, the current leader in the energy drink market [1]. More than 500 new energy drinks were launched worldwide in 2006 and beverage companies are reaping the financial rewards of the 5.7 billion dollar energy drink industry [1]. Energy drinks, including Red Bull, Amp, Monster, Rock Star, Rip It, Full Throttle, and Cocaine, are designed to give the consumer a "jolt" of energy provided by the combination of stimulants and "energy boosters" that they provide, including caffeine, herbal extracts such as guarana, ginseng, and ginkgo biloba, B vitamins, amino acids such as taurine, amino acid derivatives such as carnitine, and sugar derivatives, including glucuronalactone and ribose [1]. Energy drinks typically contain 80 to 141 mg of caffeine per 8 ounces, the equivalent of five ounces of coffee or two 12-ounce cans of caffeinated soft drink such as Mountain Dew, Coca Cola, Pepsi Cola or Dr. Pepper [2]. Energy drinks have sugar-containing and sugar-free versions. For example, Monster Energy provides 24 grams of sugar per 8 ounces (12% sugar concentration) and Rip It A'Tomic Pom provides 33 grams (14% concentration) [3,4]. Similar to the booming energy drink market, the size of the energy drink container has increased over 300-fold; Monster energy offers consumers a 23 ounces option [3].

Do energy drinks provide the consumer an extra burst of energy as the advertisements would have you believe? Yes, they do. Smit and colleagues found that energy drinks, as compared to placebo, had energizing effects among 18 to 55 year old participants, with effects being strongest 30 to 60 minutes after consumption and sustained at least 90 minutes [5]. Caffeine was found to be the primary constituent responsible for these effects. Although there is no human requirement for caffeine, even low doses of caffeine (12.5 to 100 mg) improve cognitive performance and mood [6]. However, caffeine has been found to have detrimental health consequences. Riesenhuber and colleagues found that the caffeine (but not taurine) in energy drinks promotes diuresis and natriuresis [7]. Further, acute caffeine consumption reduces insulin sensitivity [8] and increases mean arterial blood pressure [9]. High caffeine consumption is associated with chronic daily headaches, particularly among young women (age < 40 years) and among those with chronic episodic headaches and of recent onset (< 2 years) [10]. Central nervous system, cardiovascular, gastrointestinal, and renal dysfunction have been associated with chronic caffeine ingestion [11]. In sum, the caffeine in energy drinks will provide the consumer the desirable effects of increased alertness, improved memory, and enhanced mood. However, caffeine can have harmful physical consequences.

Although energy drinks are targeted to the 18 to 35 year old consumer [12], there has been little research regarding energy drink consumption patterns among young adults in the United States. The purpose of this study was to determine (1) energy drink consumption patterns among college students, (2) prevalence and frequency of energy drink use for six situations, namely for insufficient sleep, to increase energy (in general), while studying, driving long periods of time, drinking with alcohol while partying, and to treat a hangover, (3) and prevalence of adverse side effects and energy drink use dose effects among college energy drink users.

## Methods

A Registered Dietitian and a Health Educator designed a questionnaire that assessed consumption patterns of energy drinks among college students. We initially interviewed a focus group of 32 college students who were enrolled in a senior-level course. We asked these students open-ended questions regarding situations in which college students use energy drinks, the most common energy drinks college students were using, frequency patterns (average number of energy drinks consumed for each situation the focus group identified and the average number of times per month throughout a semester students use energy drinks for each situation), and side effects from using energy drinks.

Based on the focus group responses we developed a 19-item questionnaire. Questions 1 and 2 assessed demographic information (age and sex). Question 3 was a screening question, used to identify energy drink users, and asked "in an average month for the current semester do you drink more than one energy drink per month?" If a participant indicated "no", then they were instructed to skip the remaining questions in the survey and return the questionnaire to the research assistant. Participants who indicated "yes" to Question 3 were instructed to continue the survey, which assessed the type of energy drink usually consumed (regular or sugar-free), side effects associated with energy drink use (jolt and crash episodes, headaches, heart palpitations), and six situations for energy drink use (insufficient sleep, needing more energy (in general), studying for an exam or to complete a major course project, driving a car for a long time, drinking with alcohol while partying, and to treat a hangover).

For the purpose of this study, a jolt and crash episode was in reference to a feeling of increased alertness and energy (the jolt) followed by a sudden drop in energy (the crash) that occurs in response to using energy drinks.

Each of the six situation questions had two follow up questions that assessed the average number of energy drinks consumed for that situation (for example, how many energy drinks do you drink at one time following a night of not getting enough sleep?) and the average number of times per month for the current semester the student consumes energy drinks for that situation.

To provide a frame of reference regarding what constituted an energy drink, the introduction of the questionnaire included examples of energy drinks that were popular on the campus and in social establishments in the immediate geographic region when the survey was administered, these included Red Bull, Rock Star, Amp, and Full Throttle. The questionnaire was field tested among 10 randomly chosen students who were in a public location on campus. The questionnaire took approximately two minutes to complete and modifications to the questionnaire were not necessary based on the field test responses.

From mid-November to the first week of December 2006, 11 trained research assistants (undergraduate and graduate college students) recruited students at a single college from public locations across campus to participate in the study. The research assistants first ensured that those they approached were students at the university and that the student had not previously completed the questionnaire.

The institution is a state university, located in the Central Atlantic region of the United States. The fall 2006 enrollment statistics indicate an undergraduate enrollment of approximately 18,000 undergraduate and 6,000 graduate students, 85% of undergraduates were 18 to 24 years of age, 12% were 25 to 40 years of age and 3% 41 years of age and older [13]. Further, 92% of undergraduates attended school full-time whereas the majority (60%) of graduate students attended part time. In regard to ethnicity of the student body, 76% were non-Hispanic White, 16% non-Hispanic Black, 2% Asian, 2% Hispanic, 2% unknown, < 1% American Indian, and < 1% non-resident alien.. Sixty two percent of the total student body is female [13].

To diversify our sample, research assistants varied the time of day and days of the week during weekdays to recruit participants. In compliance with the university's Institutional Review Board for Research with Human Subjects (University and Medical Center Institutional Review Board number 06-0718), students were informed of the study protocol and those willing to participate anonymously completed the self-administered questionnaire. The project was carried out in compliance with the Helsinki Declaration.

Analyses were performed using JMP IN^® ^software [14]. Descriptive statistics included means, standard deviations, 95% confidence intervals, and frequency distributions. Pearson *χ*^2 ^was used to evaluate differences in frequency distribution of responses. An alpha level of .05 was used for all statistical tests.

## Results

A total of 496 participants, aged 21.5 ± 3.7 years (95% *CI *21.3, 21.8) completed the questionnaire. In regard to the first research question, energy drink consumption patterns among college students, 51% of participants (*n *= 253) reported drinking greater than one energy drink each month in an average month for the current semester, with significantly more female (53%) than male (42%) energy drink users reported, χ^2 ^(1, *N *= 496) = 6.46, *p *= .01. Seventy four percent (*n *= 187) of the 253 users drank sugar-containing versions with significantly more females (35%) than males (12%) drinking sugar-free versions, χ^2 ^(1, *N *= 247) = 16.56, *p *< .01.

Energy drink consumption patterns of college energy drink users for the six situations assessed are reported in Table 1. Insufficient sleep was the most common reason to drink energy drinks, as indicated by 67% of energy drink users. The majority of users consumed energy drinks to increase their energy (65%) and to drink with alcohol while partying (54%). Fifty percent drank while studying or completing a major course project, 45% while driving a car for a long period of time, and 17% to treat a hangover. There were no significant differences in use of energy drinks for the six situations assessed by sex, as reported in Table 1.

**Table 1:** Situations of energy drink use among college energy drink users in an average month for the current semester

Situation	% of females	% of males	χ^2^	*p *(sex)
Insufficient sleep^a^	67	68	0.17	.68
Need energy (in general)^a^	62	69	1.27	.26
Studying or major project^b^	46	56	2.22	.14
Driving car for long period of time^a^	40	51	3.01	.08
Mix with alcohol while partying^a^	57	50	1.33	.25
Treat hangover^a^	16	18	0.18	.67

^a^*n *= 146 females, 107 males, χ^2 ^(1, *N *= 253); ^b^*n *= 145 females, 104 males, χ^2 ^(1, *N *= 249).

In regard to the second research question, the percent of users drinking one, two, and three or more energy drinks by situation are reported in Table 2. The majority of energy drink users consumed one to treat a hangover, for insufficient sleep, to increase energy, and while driving a car for a long period of time. Using three or more was a common practice (49% of users) to drink with alcohol while partying. The percent of users drinking energy drinks 1 – 4, 5 – 10, and 11 or more days in an average month for the current semester are also reported in Table 2. For the six situations assessed, the majority of users (73% to 86%) consumed energy drinks 1 – 4 days in a month.

**Table 2:** % of college energy drink users^a ^reporting amount and frequency of energy drink consumption by situation in an average month for the current semester

		Energy drinks consumed			Days per month		
		
Situation	*n*	1	2	≥3	1 – 4	5 – 10	≥11
Insufficient sleep	169	64	22	14	74	18	8
Need energy (in general)	165	63	21	16	74	18	8
Studying or major project	125	50	36	14	85	10	5
Driving car for long period of time	114	63	23	14	86	9	5
Mix with alcohol while partying	136	27	24	49	73	18	9
Treat hangover	42	74	10	16	74	14	12

^a^*n = *253 college energy drink users.

To further identify relationships between the six situations of energy drink use and energy drink consumption patterns, we summed the number of situations for reported energy drink use and compared this to the maximum number of energy drinks consumed for any of the six situations. These results are reported in Table 3. By sum of situation categories, 16% to 20% of energy drink users consumed energy drinks for a total of one to five of the six situations, 7% consumed energy drinks for a total of all six. As total situations increased so did the maximum energy drink consumption for at least one situation. For example, 40% to 81% of energy drink users who reported a total of three or more situations consumed three or more energy drinks for at least one situation, whereas 29% of those with a total of one situation and 18% of those with a total of two consumed three or more for at least one situation.

**Table 3:** Maximum number of energy drinks consumed for any of six situations by sum of situations^a ^in an average month for the current semester among college energy drink users

		Maximum energy drink intake for any situation		
		
Parameter	*n*	1	2	≥3
% of 1 situation responders	45	56	15	29
% of 2 situation responders	38	55	27	18
% of 3 situation responders	45	33	27	40
% of 4 situation responders	46	20	37	43
% of 5 situation responders	43	5	32	63
% of 6 situation responders	16	6	13	81

Note: χ^2 ^(10, *N *= 233) = 53.43, *p *< .01.

^a^Sum of six situations reported energy drink use.

Regarding the third research question, weekly jolt and crash episodes were experienced by 29% of users, 22% reported ever having headaches and 19% heart palpitations from consuming energy drinks, which did not differ significantly by sex, χ^2 ^(1, *N *= 253) < 0.01, *p *= .97 for jolt and crash episodes, χ^2 ^(1, *N *= 234) = 0.37, *p *= .54 for heart palpitations, χ^2 ^(1, *N *= 252) = 0.45, *p *= .50 for headaches. The data for side effects by energy drinks consumed are reported in Table 4. There was a significant dose effect for jolt and crash episodes but not for heart palpitations or headaches. For example, 57% of energy drink users who reported experiencing weekly jolt and crash episodes also consumed three or more energy drinks for at least one situation, whereas 35% of those who denied jolt and crash episodes consumed three or more.

**Table 4:** Side effects by maximum number of energy drinks consumed for any of six situations in an average month for the current semester among college energy drink users

		Maximum energy drink intake				
Parameter	*n*	1	2	≥3	χ^2^	*P *(yes versus no)
Weekly jolt and crash episodes	253				19.10	< .01
% of yes responders	74	12	31	57		
% of no responders	159	40	25	35		
Ever having heart palpitations	215				4.77	.09
% of yes responders	40	20	22	58		
% of no responders	175	34	27	39		
Ever having headaches	232				1.24	.54
% of yes responders	51	27	24	49		
% of no responders	181	33	27	40		

## Discussion

Energy drinks are marketed to young adults and marketing efforts may be particularly appealing among college students. For example, Cocaine energy drink, with a Cut Cocaine variety, has been marketed as a "legal alternative" to the class A drug [15]. On April 4, 2007, the Food and Drug Administration issued a warning to Drink Reboot, the firm that markets Cocaine, citing numerous marketing violations, including promoting this product as a street drug alternative [15]. Red Bull energy drink is reportedly a "functional beverage" that was designed to increase physical and mental performance and "is appropriate to drink during sports, while driving, and during leisure activities" [16] whereas Monster energy provides a "double shot of our killer energy brew. It's a wicked mega hit that delivers twice the buzz of a regular energy drink..." [3]. The purpose of this study was to identify energy drink consumption patterns and side effects associated with consumption of energy drinks among college students. We found that energy drink consumption is a popular practice among college students, particularly if the student has had insufficient sleep, if they need more energy in general, while studying for exams or working on major course projects and while driving an automobile for a long period of time.

Improvements in mental functioning are of interest among college students, many who suffer from sleep deprivation. The American College Health Association reported that 71% of college students whom they surveyed reported insufficient sleep and not feeling rested for at least five of the past seven days [17]. Sleep deprivation is associated with selecting less difficult cognitive tasks and college students who have sleep difficulties report a greater frequency of stress [18,19]. Findings from our study support the premise that college students use energy drinks to treat sleep deprivation and while studying for exams or completing major course projects. On the other hand, caffeine consumption has not been found to affect academic performance among college students [20].

The primary ingredient in energy drinks that has a cognitive stimulating effect is the caffeine [5], whereas high sugar content (18% concentration) does not improve reaction times slowed by sleep deprivation [21]. Further, the combination of caffeine and taurine has no effect on short-term memory [9]. Although low doses of caffeine (12.5 to 50 mg) have been found to improve cognitive performance and mood [6] and 200 mg doses have been found to improve cognitive task speed and accuracy and increase alertness among young adults [22], the amount of caffeine provided in energy drinks can easily far exceed the amount necessary to promote cognitive functioning [23]. This is especially true if a student is consuming 16- or 23-ounce cans or multiple cans of energy drinks for a given situation. Although we did not assess the size of the energy drink cans that participants normally consumed, results from our study indicate that in some situations while students are consuming energy drinks, the amount of caffeine that they consume can exceed the amount needed simply to promote cognitive stimulation. For example, 50% of energy drink users in our study drank two or more energy drinks while studying for an exam or working on a major course project, and 36% to 37% drank two or more following insufficient sleep, when they needed energy throughout the day, or while driving an automobile for a long period of time. Further, drinking multiple energy drinks with alcohol was a popular practice among 73% of energy drink users. The practice of consuming greater amounts of caffeine while socializing has also been documented among American youth [24] and an alcoholic setting is considered by many college students a primary locus to socialize and to meet people [25].

Results from the present study indicate that female and male college students are using energy drinks in a similar fashion. Whereas we found a greater prevalence of energy drink consumption and greater use of sugar-free varieties of energy drink use among females, we identified no situation differences nor prevalence of side effects from consuming energy drinks between sexes.

There are a number of limitations to this study that deserve discussion. First, in an effort to ensure the survey instrument could be completed quickly, we collected limited demographic information. Based on the descriptive statistics regarding age, we primarily had undergraduate participants and a slightly greater percentage of male participants as compared to the sex distribution at the university. On the other hand, random sampling throughout the weekdays and times of the day at central locations throughout campus was an advantage to the study design. Additionally, this is a rural state university with a fairly homogenous student body. Second, the data collected was self-reported. In particular, frequency patterns of energy drink intake were asked by situation and were treated as independent and distinct events, which may not have been the case. For example, and energy drink user may consume energy drinks because they had not gotten enough sleep and because they were studying for an exam. As a result, assessment of energy drink consumption may have been overestimated for each of the situation events. On the other hand, the results from this study provide important and novel information regarding energy drink consumption habits among college students. Of particular importance is the finding that using energy drinks for a number of situations is common among college students and that those who use energy drinks for three or more of the situations that we assessed tended to drink three or more energy drinks for at least one situation. Further, side effects of jolt and crash episodes, heart palpitations, and headaches are fairly common, as reported by approximately 25% of users, and there is a significant dose effect of energy drink consumption and jolt and crash episodes.

## Conclusion

Using energy drinks is a popular practice among college students, as we found that 51% of 496 college students surveyed reported drinking greater than one energy drink each month. Among college energy drink users, consuming energy drinks is particularly popular for insufficient sleep, when one needs more energy in general, to drink with alcohol while partying, and when studying for an exam or completing a major course project. Drinking three or more for a given situation occurs more frequently among those who consume energy drinks for three or more of the six situations that were assessed. Side effects of consuming energy drinks, including experiencing jolt and crash episodes, hear palpitations, and headaches occur in many energy drink users. However, a dose effect was found only for jolt and crash episodes. Further research should identify if students recognize the amounts of caffeine that are present in the wide variety of caffeine-containing products they consume, the amounts of caffeine that are consumed in various situations, and the physical side effects associated with caffeine consumption.

## Competing interests

The author(s) declare that they have no competing interests.

## Authors' contributions

BMM participated in the study design, performed the statistical analysis, and drafted the manuscript. VGA conceived of the study and drafted the manuscript. AJC assisted with statistical analysis and helped to draft the manuscript. RFO, TCA, and KBH participated in coordination and data collection and helped to draft the manuscript. All authors read and approved the final manuscript.
